# Genetic Mosaics and the Germ Line Lineage

**DOI:** 10.3390/genes6020216

**Published:** 2015-04-17

**Authors:** Mark E. Samuels, Jan M. Friedman

**Affiliations:** 1 Department of Medicine, University of Montreal, Montreal, QC H3T 1C5, Canada; 2 Centre de Recherche du CHU Ste-Justine, Montreal, QC H3T 1C5, Canada; 3 Department of Medical Genetics, University of British Columbia, Vancouver, BC V6H 3N1, Canada; E-Mail: jfriedman@cfri.ca

**Keywords:** mosaicism, germ line, lineage, *de novo* mutation

## Abstract

Genetic mosaics provide information about cellular lineages that is otherwise difficult to obtain, especially in humans. *De novo* mutations act as cell markers, allowing the tracing of developmental trajectories of all descendants of the cell in which the new mutation arises. *De novo* mutations may arise at any time during development but are relatively rare. They have usually been observed through medical ascertainment, when the mutation causes unusual clinical signs or symptoms. Mutational events can include aneuploidies, large chromosomal rearrangements, copy number variants, or point mutations. In this review we focus primarily on the analysis of point mutations and their utility in addressing questions of germ line *versus* somatic lineages. Genetic mosaics demonstrate that the germ line and soma diverge early in development, since there are many examples of combined somatic and germ line mosaicism for *de novo* mutations. The occurrence of simultaneous mosaicism in both the germ line and soma also shows that the germ line is not strictly clonal but arises from at least two, and possibly multiple, cells in the embryo with different ancestries. Whole genome or exome DNA sequencing technologies promise to expand the range of studies of genetic mosaics, as *de novo* mutations can now be identified through sequencing alone in the absence of a medical ascertainment. These technologies have been used to study mutation patterns in nuclear families and in monozygotic twins, and in animal model developmental studies, but not yet for extensive cell lineage studies in humans.

## 1. Embryonic Origin of the Mammalian Germ Line

The cells that give rise to the germ line in humans are set aside early during embryogenesis [1]. The first cells that have been identified in humans as contributing to the germ line are progenitor germ cells (PGCs), which are distinguished by their specific cellular morphology and high-level expression of alkaline phosphatase [2,3,4]. In human embryos, as few as 30–50 PGCs have been observed in 12–13 somite embryos (Carnegie stage 10–11, 28–30 days post-fertilization) [5,6]. In 7–8 day old mouse embryos, counts of 10–150 PGCs have been made [5,7]. In both humans and mice, PGCs are first recognizable in the dorsal wall of the yolk sac near the developing allantois, and migrate through the embryo, eventually arriving near the endodermal yolk sac, where they are incorporated into the surrounding gonadal tissue developing from somatic cells [1,2,8,9,10]. A number of molecular markers, including *VASA*, *DAZL*, *NANOG*, *c-KIT*, and *POU5F1*, have been described in human embryonic germ cells*;* however, it is unclear whether these markers are all expressed in the earliest PGCs [10,11,12,13,14].

The earliest stages of germline specification are poorly understood in humans. In mice it has been shown that six cellular precursors to PGCs (called founding germ line cells or FGCs) originate in the epiblast, and, in response to bone morphogenetic protein signalling from surrounding cells, express *Smad1-5*, *Prdm1* (also known as *Blimp1*) and *Prdm14*. The FGCs subsequently migrate to the extraembryonic region, where they proliferate and develop into PGCs with expression of *Stella*, *Kit*, *TNAP* and *SSEA1* [15]. Given differences in early mouse and human embryonic development, it is not clear whether the same series of events occurs in human FGC specification [15]. Combining cell counts and theoretical modelling of cell division rates, Zheng *et al.* suggested that there may be as few as 2–3 FGCs in humans [16]. However, their analysis does not address the clonality of FGCs [1]. Here the term “clonal” is used with a specific sense, meaning that all cells of the eventual germ line derive from a single ancestral cell, whose descendants include only germ line and not somatic tissue. Thus, the fertilized egg, while being the ultimate ancestral cell of the entire embryo, is not a clonal ancestor of the germ line because its descendants also include soma and extra-embryonic tissues.

In principle, there are several possibilities for initial specification of the germ line. A single embryonic cell, or all descendants of such a cell, might become the first FGC (henceforth referred to as FGC_1_), with the entire germ line descended from that/those cell(s). Alternatively, two or more cells with different ancestries might independently be recruited as FGCs (henceforth referred to as FGC_1,2,3,…_). In humans, these alternatives are impossible to resolve through histological observation alone, given the narrow time windows, the small number of cells involved at the earliest stage of germ line recruitment, and the very limited opportunity to examine human embryos at exactly the right point in development.

The use of cytogenetic or molecular genetic mosaicism to study germ line lineage, which was proposed in detail more than 40 years ago by Nesbitt and Gartler among others [17], has not been widely exploited in humans. Such mosaics arise through post-fertilization *de novo* mutation leading to genetically mixed cell populations in an embryo derived from a single fertilized egg, and are well-documented in humans [18,19,20,21]. Germ cell development has been studied using spontaneous and mutagen-induced mosaicism in mice, providing evidence of a polyclonal origin of the germ line in both males and females [22]. In another approach, animals such as mice or chicks can also be created as chimeras having mixed cell populations by physically merging two or more embryos with different genetic backgrounds. Such physical chimeras have been observed as rare spontaneous events in humans, arising through fusion in multi-embryonic pregnancies [23,24].

## 2. Germ Line and Zygotic *de novo* Point Mutation Rates

Recent sequencing studies using next-generation sequencing technology have documented that each individual’s genome contains at least 50–100 new point mutations that were not present in the genomes of either parent [25,26,27,28,29,30,31]. These are referred to as *de novo* mutations. Here, point mutations include single nucleotide variants (SNVs) as well as small insertions and deletions (indels). This is likely an underestimate of the total mutation rate as repetitive sequences are difficult to analyse by next-generation sequencing; hence mutations arising in these genomic regions may be underrepresented although rates in these regions may be higher than average due to difficulties during replication or recombination. Larger copy number variants (CNVs) and other structural variants (SVs) such as chromosomal rearrangements are also excluded from this total, although such mutations have been documented and probably occur in most zygotes [31].

Interpretation of apparent *de novo* mutation rates requires consideration of the source of DNA used for the analyses. Most genome sequencing studies are performed using DNA extracted from peripheral blood cells. Such DNA is obtained from nucleated leukocytes in blood, comprising a variety of hematopoietic cell types whose relative proportions vary and depend on the specific method of separation used. Lymphocytes comprise the major cell type remaining after Ficoll separation, thus the term lymphocyte DNA is frequently used in the genetics literature. Here the more general term leukocyte will be used. The issue of DNA source is relevant for mosaicism studies. The measured rate of 50–100 *de novo* point mutations per zygote, observed from sequencing leukocyte DNA, includes two separate classes of events: mutations arising in a parental germ cell or germ line precursor, or mutations arising post-fertilization in the offspring. The latter events are usually called somatic mutations, although if they arise early enough during embryogenesis, they could be present in both the soma and germ line of the offspring. These two classes of events, parental germ line and zygotic mutations, cannot be distinguished simply from analysis of leukocyte DNAs. Additional studies are required as described below.

The relative contribution of parental *versus* zygotic *de novo* mutation events can be estimated by comparing the genomes of monozygotic twins. Genomic differences shared by both twins and neither parent represent pre-twinning events, of either post-fertilization but very early zygotic or else parental germ line origin. Genomic differences between MZ twins reflect mutations arising post-twinning. Large-scale chromosomal rearrangements, aneuploidy and copy number variant (CNV) differences between MZ twins have been well-documented for quite some time [32]. More recently, studies using whole genome sequencing or high density SNP genotyping have demonstrated the occurrence of post-twinning point mutations. These are very rare, with at most a few per twin pair based on comparing leukocyte DNA. Thus most of the 50–100 *de novo* point variants found by comparing parental and offspring leukocyte DNA are the result of mutations occurring either in the parental germ lines or in the zygote before twinning occurs, which may be as late as the gastrula stage 9–12 days after conception [25,33,34,35,36,37,38,39,40,41,42]. The relatively small size of such early zygotes suggests that most observed *de novo* events probably occur in a parent. As an aside, repetitive genomic regions notwithstanding, most *de novo* variants fall in non-protein coding regions as coding exons comprise only approximately 1.5% of the haploid genome. Indeed, rates of deleterious *de novo* mutation leading to dominant genetic disorders are substantially lower than the total rate of *de novo* mutations. *De novo* mutations within coding regions occur at most a few times per zygote, and about half of these are synonymous and thus generally without deleterious effects. Despite such a low rate of new deleterious mutations per live birth, the human protein-coding gene repertoire is saturated with deleterious mutations in the more than 130 million of live births that occur worldwide annually, although most are expected to be silent due to their recessive effects [43,44,45].

The general rarity of *de novo* mutation events has until recently made the ascertainment and study of such events difficult, except when there is a significant medical impact (although the advent of next-generation sequencing technologies will mitigate against the rarity of these events in future studies). Their rarity also implies that most such events will generate a variant in the heterozygous state in diploid cells (except on the X or Y chromosome in males). Although many or perhaps most deleterious mutations in the human genome behave as recessive alleles, some mutations do behave dominantly to cause particular genetic disorders; a subset of these may result in medical disorders even if present in only some somatic cells [45]. In such cases, clinical ascertainment may allow the identification of *de novo* mutational events in probands with genetic disorders associated with high penetrance heterozygous mutations, whose parents do not share the disorder [18,21]. Many studies have documented such examples, which are usually demonstrated by direct sequencing of the causal gene (if known or suspected), or else by whole genome or whole exome sequencing. In some disorders *de novo* dominant mutations are the main source of new cases, if the disorder results in reduced viability or reproductive fitness.

## 3. Mosaicism Patterns Depending on Germ Line Lineage

**Figure 1:**
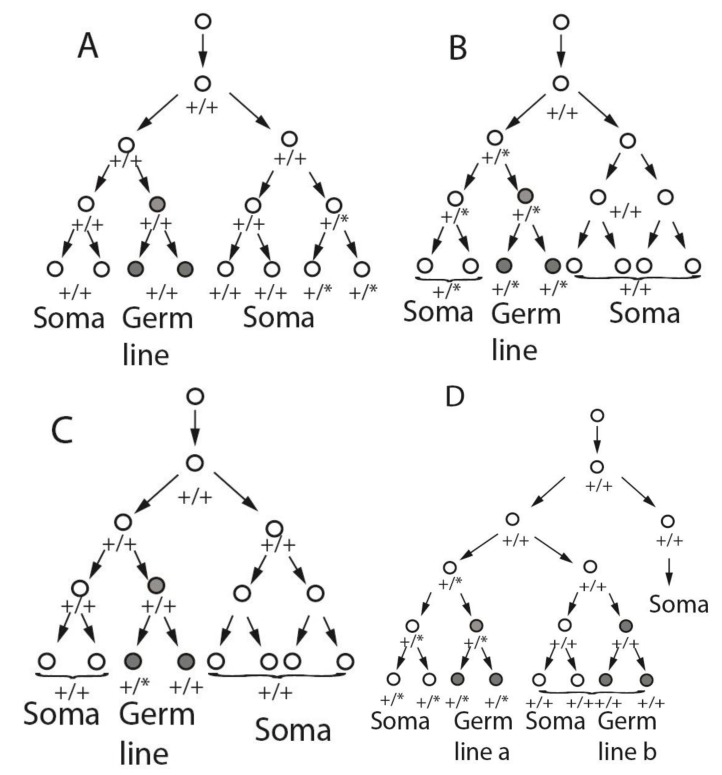
Potential cell lineages of somatic and germ line mosaicism based on alternative specification scenarios. In each panel, the one cell zygote is shown at the top, and following a small number of divisions. At some point, a wild type (+/+) cell gives rise to a mutant cell (+/*), which divides to produce a set of descendants that include all or part of the germ line and/or part of the soma. Panels (**A**), (**B**), and (**C**) assume that a single post-zygotic cell (FGC_1_) gives rise to the entire germ line. Panel (**D**) assumes two or more post-zygotic cells, of different post-fertilization ancestry, contribute to different portions of the germ line (Germ line a, Germ line b). In each panel, cells contributing to the germ line are shaded in grey. *De novo* mutations are indicated as asterisks so that cells carrying the mutation are heterozygous +/*. Panel (**D**) shows the lineage outcome in the event of mutation in a cell that gives rise to both germ-line and somatic descendants, in the case of multiple (*i.e.*, non-clonal) germ line precursors. These trees are not meant to reflect specific actual cell lineages or the relative size of germ line *versus* somatic cell compartments.

**Figure 2:**
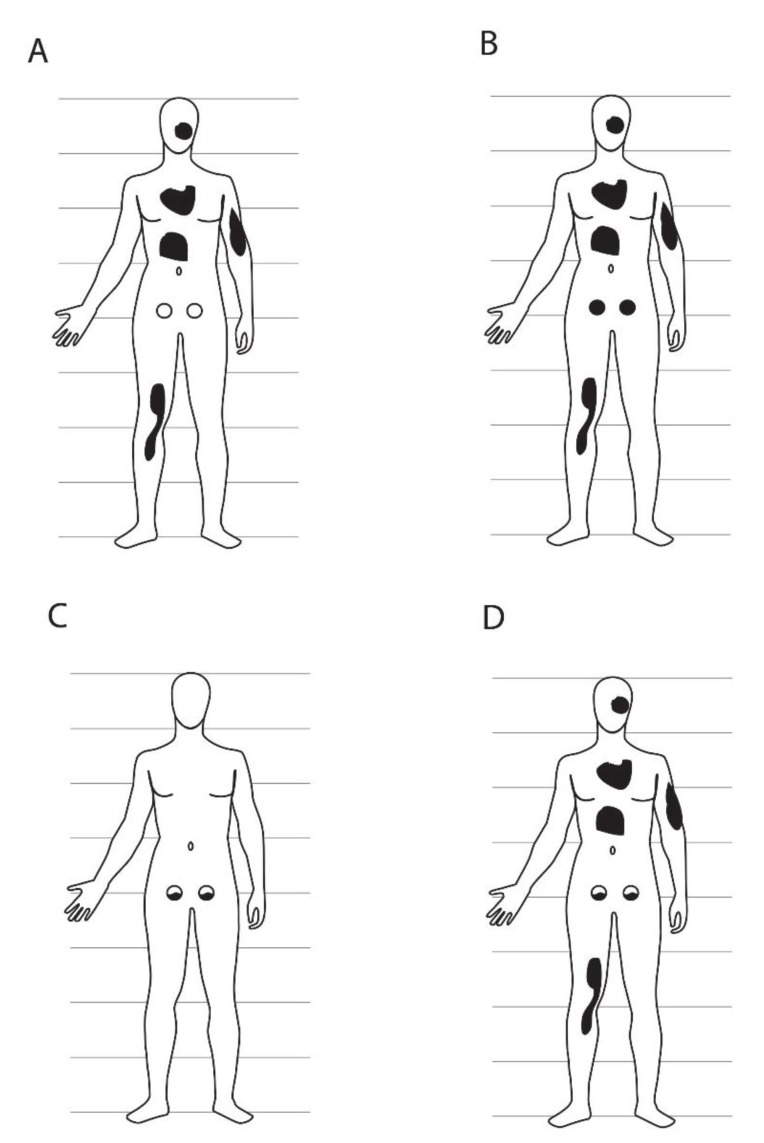
Potential anatomical patterns of mosaicism based on the lineage and mutational scenarios of Figure 1. Each panel is matched to the equivalent panel in Figure 1 (panel **A**, germ line clonal, mutation in soma; **B**, germ line clonal, mutation in cell ancestral to germ line and some of soma; **C**, germ line clonal, mutation in germ line; **D**, germ line not clonal, mutation in cell ancestral to some of germ line and some of soma). The germ line is presented as two circles in the lower abdomen, either wild type (unshaded) or mutant (black). Patches of somatic tissue carrying a *de novo* mutation are shown as irregular black shapes and may include skin and/or any other kind of somatic cells. In panels (**C**) and (**D**), germ line mosaicism is shown in both the right and left gonad, but other gonad patterns are likely as well, depending on how the primordial gonads are populated by PGCs. Relative sizes of the germ line *versus* soma are not to scale; the germ line in humans is obviously much smaller than the soma in physical size and cell number. Patterns of mosaicism are also not meant to be interpreted literally, other than inclusion or exclusion of all or part of germ line *versus* soma.

The occurrence of germ line and somatic mutations provides an opportunity to explore the early germ line lineage. Consider a new mutation arising in some cell during embryogenesis. Assuming that the mutation is not deleterious at the cellular level, this cell and all of its descendants will be heterozygous for the mutation (or hemizygous in the case of X- or Y-linked non-pseudo-autosomal mutations in males). If these descendants include only somatic cells, then various somatic tissues will carry the mutation in various proportions, depending on exactly when and where in the embryo the mutation arose (Figure 1A). The individual will be a somatic mosaic, with a completely normal germ line (Figure 2A). The lack of access to most tissues in living humans makes comprehensive studies of such somatic mosaicism difficult, although inter-tissue variation of mutant cell frequencies has been observed by analyzing multiple tissues that can be sampled routinely (blood, hair follicles, buccal epithelium, urine, or skin fibroblasts) [46]. One example with medical ascertainment is Proteus syndrome, in which mosaic mutations of the *AKT1* gene are found in multiple tissues but rarely in hematopoietic cells [47,48]. Another extensively studied disorder is McCune-Albright syndrome, in which mosaic mutations in the *GNAS1* gene are found in multiple endocrine and non-endocrine tissues, leading to the clinical phenotype [49,50]. Many cancers depend on somatic mutations arising in particular embryonic (or post-embryonic) cells at the right time and place to result in the particular phenotype.

Alternatively, a mutation might arise in a cell whose descendants include both soma and germ line (Figure 1B). In this case, if there is only a single clonal FGC_1_, the soma would be mosaic, whereas germ line pre-meiotic cells would be completely mutant (and heterozygous (excepting the non-pseudo-autosomal X or Y in males) prior to meiosis, Figure 2B). Post-meiotic gametes would be either wild type or mutant, in equal ratio.

In the third case, a mutation might arise in a mitotic germ line descendant of the initial FGC(s) (Figure 1C), resulting in a mosaic germ line and completely wild type soma (Figure 2C). Such mutations could be transmitted to multiple gametes derived by meiosis from different mitotic descendants of the initially mutated cell over the course of years. Participation of those gametes in fertilization would lead to multiple cases in of the same, apparently *de novo* genetic disorder, recurrent in the family but not through multiple independent mutational events. Such families have been repeatedly, albeit infrequently, reported in the medical literature. The potential for such intra-familial recurrence is often overlooked by non-geneticists, and is particularly relevant for genetic counsellors attempting to estimate recurrence risks in families. The recurrence risk in these cases could range from effectively nil (in the case of an immediately pre-meiotic mutation leading to only a single carrier gamete) to almost 50% (in the case of a heterozygous mutation arising early in germ line clonal mitotic expansion and hence expected in a large proportion of gametes). Some such mutations appear to provide a selective advantage to the mitotic germ line cells in which they occur, which can further bias the expected intrafamilial recurrence risk [51,52,53,54].

The single FGC_1_ hypothesis–that all germ cells are clonally derived from a single progenitor cell that gives rise to all of the germ line and no somatic cells–is consistent with all of the outcomes regarding post-zygotic *de novo* mutations shown in Figure 1A–C. However, this hypothesis is ***not*** consistent with the outcome shown in Figure 1D and Figure 2D, in which both the soma and germ line are mosaic for the same mutation. This outcome is only possible if the germ line is derived from two or more germ line-specific progenitor cells, only one of which is a descendant of the originally mutated cell.

Here an important point of terminology must be made. In the clinical genetics literature, germ line mosaicism is inferred in families in whom there are two or more children with the same fully penetrant autosomal dominant disease that is not obviously present in either parental genome. The term “germ-line mosaicism” as used in the literature thus does not necessarily imply *exclusive* germ-line involvement; somatic mosaicism may or may not also be present. In some clinical reports of germ-line mosaicism, the mutation has been demonstrated in a proportion of the affected parent’s germ cells (usually the father’s sperm, see examples in Table 1). In many more instances, the mutation has been tested for and may or may not have been found in the parents’ blood and/or other somatic tissues. In this discussion, we use the term “germ line mosaicism” to mean that a portion, but not all, germ cell precursors (or germ cells) in a given individual have a particular mutation; mosaicism or lack thereof, in parental somatic tissue is not automatically implied. It should be noted that a definitive negative interpretation of parental somatic tissue, expected in the case of pure germ line mosaicism, is difficult to prove given the limited access to most somatic tissues.

If the germ line arises from two or more FGCs with different clonal histories, then combined somatic and germ line mosaicism (Figure 1D and Figure 2D) is possible. The situations depicted in Figure 1A–C are also consistent with multiple FGCs, with probabilities that depend on embryonic timing and location of mutations and developmental fate of the mutant cells, but demonstration of mosaicism for the same mutation in both the germ line and somatic tissues of an individual is only compatible with multiple FGCs that are *not* clonally closely related to each other.

## 4. Observational Tests of Combined Germ Line and Somatic Mosaicism

There are several ways to assess the mosaic status of the soma and germ line. The simplest observations are cytogenetic in the case of a chromosomal aneuploidy or major rearrangement. Molecular approaches may involve measuring the ratio of wild type to mutant alleles in either compartment. In a sample of fully mutant heterozygous cells with no mosaicism, this ratio should be 1/1, *i.e.*, 50% mutant and 50% normal or reference allele. In the event of mosaicism, this proportion may be less than 50%, depending on what fraction of the tissue sample includes descendants of the initially mutant cell. The proportion of alleles that are mutant can be determined by Sanger sequencing and quantifying the ratio of normal to mutant allele peak heights in sequencing chromatograms at the variant site, or by counting PCR clones of amplified fragments containing the mutation, or by counting individual normal and mutant reads at the variant site following next-generation whole genome or exome sequencing. Alternatively, the two alleles can be quantified by differential hybridization or quantitative PCR in a custom genotyping assay. Somatic mosaicism can also be studied in different somatic tissue sources as noted above.

**Table 1:** Candidate examples of combined somatic and germ line mosaicism in genetic disorders.

Disorder and Clinical Status of Affected Offspring	Gene and Chromosome	Somatic Mosaicism in Parent *	Germ Line Mosaicism in Parent *	Mosaic Parent and Clinical Status
X-linked dominant protoporphyria [55], One severely affected	*ALAS2* ChrX	Yes Sequencing, 13% mutant allele in peripheral blood and buccal mucosa	Yes Aff/unaff half-sibs share same maternal X haplotype with/without mutation	Mother Mildly affected
Androgen insensitivity [56] Two siblings differentially affected, one raised as female one as male	*AR* ChrX	Yes Allele specific oligo hybridization <10% mutant allele in peripheral blood	Yes Aff/unaff sibs share same maternal X haplotype with/without mutation	Mother Unaffected
Osteogenesis imperfecta [57] Two half-siblings, one deceased pre-term, one deceased neonatally	*COL1A1* Chr17	Yes RFLP, 20% mutant allele in peripheral blood, hair bulbs, absence of mutant allele in fibroblasts	Yes RFLP, 14% mutant allele in sperm	Father Unaffected
Osteogenesis imperfecta [58] One deceased perinatally	*COL1A1* Chr17	Probably Allele-specific hybridization, variable proportion mutant allele 26% in peripheral blood 45%–50% in fibroblasts	Probably Allele-specific hybridization, 36%–40% mutant allele in sperm	Father Mildly affected
Osteogenesis imperfecta [59] Two siblings, one more severe deceased at 3 years	*COL1A1* Chr17	Yes Allele specific hybridization, library colony count, variable proportion mutant allele in peripheral blood (~10%), fibroblasts (~25%)	Possibly Allele specific hybridization, library colony count, 40%–45% mutant allele in sperm	Father Unaffected
Osteogenesis imperfecta [60] Two half-siblings, severely affected deceased neonatally	*COL1A2* Chr7	Probably Southern blot, variable stoichiometry of mutant allele in peripheral blood (40%), fibroblasts (almost 50%)	Probably Southern blot, 40% mutant allele in sperm	Father Moderately affected
Osteogenesis imperfecta [61] One affected proband, two deceased prenatally	*COL1A2* Chr7	Yes 25% mutant allele in peripheral blood, fibroblasts	Possibly 40% mutant allele in sperm	Father Unaffected
Rubinstein-Taybi syndrome [62] One significantly affected (FII)	*CREBBP* Chr16	Possibly Sanger sequencing, small secondary peak of mutant allele in saliva, blood (not quantified)	Possibly Sanger sequencing, small secondary peak of mutant allele in sperm (not quantified)	Father Very mildly affected if at all
Dyskeratosis congenital [63] Two affected males, two unaffected mutation carrier het females with skewed X-inactivation	*DKC1* ChrX	Yes Allele-specific PCR, mutant allele observed but <5% in peripheral blood, saliva	Yes Aff/unaff brothers share same maternal X haplotype with/without mutation	Mother Unaffected
Duchenne muscular dystrophy [64] Family DL114, one affected proband	*DMD* ChrX	Possibly Southern blot, mutant allele band less than 50% in peripheral blood	Yes Aff/unaff sibs share same maternal X haplotype with/without mutation	Mother
Duchenne muscular dystrophy [65] Proband hemizygous, severely affected from age 3 years	*DMD* ChrX	Yes Microsatellite genotyping and PCR deletion detection Three alleles detected in maternal lymphocytes	Yes Aff/unaff brothers share same maternal X haplotype with/without mutation	Mother Unaffected
Haemophilia B [66] One severely affected hemizygous male of heterozygous mother, mosaicism analyzed in her father (“grandfather”)	*FIX* ChrX	Yes DHPLC, 35% mutant allele in peripheral blood	Yes Aff/unaff half-sisters share same grandpaternal X haplotype with/without mutation	Grandfather Mildly affected in clotting assay
Facioscapulohumeral muscular dystrophy [67] One affected in each of two families (F4, F13)	*FSHD1 Chr4*	Probably Southern blot Signal of mutant *versus* normal RFLP band in peripheral blood lower than in non-mosaic affected progeny (semi-quantitative)	Yes Southern blot Aff/unaff sibs share same maternal haplotype with/without mutation in both families	Mothers (2 families) Possibly affected
Haemophilia A [68] One affected male proband, mutation from mosaic mother	*FVIII* ChrX	Possibly Southern blot, causal mutant allele much less than 50% in peripheral blood	Possibly Southern blot, three gene alleles among progeny	Mother Unaffected
Haemophilia A [69] One affected male proband, mutation from heterozygous unaffected mother, mosaic was maternal grandfather	*FVIII* ChrX	Yes Sequencing and PAGE, normal and mutant allele of X-linked gene present in peripheral blood, buccal cells	Yes Sequencing and PAGE, 2 sisters of proband’s mother, normally obligate mutation carriers, lacked mutant allele	Grandfather Unaffected
Lesch-Nyhan syndrome [70] Proband hemizygous for mutation, undiagnosed brother deceased age 1 month	*HPRT1* ChrX	Yes Cultured cell clones with or without mutation	Yes Aff/unaff sisters share same maternal X haplotype with/without mutation (heterozygous)	Mother Unaffected
Hunter disease [71] Proband hemizygous for mutation	*IDS* ChrX	Yes Allele-specific hybridization, quantitative PCR, variable mutant allele frequencies 7% in lymphocytes, leukocytes, 22% in fibroblasts, 1/35 hair bulbs	Yes Aff/unaff brother/sister share same maternal X haplotype with/without mutation	Mother Unaffected
CRASH syndrome [72] Proband hemizygous for mutation, mother heterozygous carrier, one affected hemizygous uncle	*L1CAM* ChrX	Yes RFLP, SSCP, mutant allele signal less than in true heterozygotes in family.	Yes Aff/unaff siblings share same grandmaternal X haplotype with/without mutation	Grandmother Unaffected
Neurofibromatosis [73] Mosaic mother is proband, affected daughter simple heterozygote for mutation	*NF2* Chr22	Yes Quantitative Sanger sequencing, 18% mutant allele in peripheral blood	Yes Aff sister/unaff brother share same maternal haplotype with/without mutation	Mother Affected diagnosed age 23 years
Lowe syndrome [74] One affected hemizygous mutation carrier in family LS04FR, heterozygous unaffected mother	*OCRL* ChrX	Possibly Single strand conformation analysis, small proportion of mutant allele detected in urine, none in blood, buccal or hair bulb	Yes 1 carrier, 2 normal sisters share same grandmaternal X haplotype with/without mutation	Grandmother Unaffected
Hypophosphatemic rickets [75] One affected diagnosed age 19 months, 56% mutant allele as per simple heterozygote	*PHEX* ChrX	Yes Single-base extension and DHPLC, 60% mutant allele in lymphocytes, 6%–94% mutant allele in multiple independent hair bulbs	Yes Aff/unaff sisters share same paternal X haplotype with/without mutation (heterozygous)	Father Affected, treatment initiated age 2 years, grandparents unaffected
Polycystic kidney disease [76] One affected diagnosed age 17 years	*PKD1* Chr16	Yes Next-generation sequencing, 3% mutant allele in peripheral blood, 4% in buccal cells (below detection limit by Sanger sequencing)	Yes Sanger, next-generation sequencing, 10% mutant allele in sperm	Father Affected, diagnosed age 50 years
Alzheimer disease [77] Mosaic mother is proband, onset age 42 years, deceased age 58 years. One daughter inherited mutation fully heterozygous, more severe, onset age 27 years, deceased age 39 years	*PSEN1* Chr14	Yes Allele-specific hybridization, mutant allele in peripheral blood, autopsy cerebral cortex much lower signal than in heterozygous daughter (qualitative), mutant detected by sequencing cerebral cortex but not peripheral blood DNA	Yes 1 aff/2 unaff sibs share same maternal haplotype with/without mutation	Mother Affected
Retinoblastoma [78] Three families (139, 345, 385) each with one bilaterally affected proband	*RB1* Chr13	Yes PCR SSCP, mutant allele less than 50% in peripheral blood in all three mosaic parents	Yes Aff/unaff sibs share same parental haplotype with/without mutation (all 3 families). In one family, mutation observed in 20%–30% of father’s sperm	Father (two families) Mother (one family) All unaffec ted
Retinoblastoma [79] Families D, E one bilaterally affected proband in each	*RB1* Chr13	Yes RFLP, sequencing individual PCR clones from peripheral blood, 10% clones mutation positive in fam D, 12% in fam E. Single-sperm PCR RFLP, 7% mutation-carrying sperm in fam E	Yes Aff/unaff sibs or half-sibs share same paternal haplotype with/without mutation	Fathers (two families, one bilaterally, one unilaterally affected)
Spinal muscular atrophy [80] One affected inheriting mutation independently from both parents, father het carrier, paternal grandmother is candidate mosaic	*SMN1 Chr5*	Possibly Microsatellite genotyping showed 3 chr5 haplotypes, qPCR showed intermediate gene dosage in peripheral blood	Possibly Affected/unaffected progeny share same grandmaternal haplotype with/without mutation	Grandmother Unaffected
Anophthalmia syndrome [81] One severely affected, second deceased pre-term not studied	*SOX2* Chr3	Yes RFLP by DHPLC, mutant allele present with lower signal in blood, mouthwash of parent than in non-mosaic affected heterozygous offspring (qualitative)	Yes Aff/unaff sibs share same maternal haplotype with/without mutation	Mother Unaffected
46,XY disorder of sexual development [82] Two fully sex-reversed XY siblings	*SRY* ChrY	Yes Normal and mutant SRY alleles seen for Y chromosome in peripheral blood (qualitative)	Yes Normal and mutant SRY alleles seen for Y chromosome in sperm (qualitative)	Father Unaffected

Abbreviations: “Chr” chromosome, “Oligo” oligonucleotide, “Aff/unaff” affected/unaffected, “Fam” family, “RFLP” restriction fragment length polymorphism, “SSCP” single strang conformation polymorphism, “PAGE” polyacrylamide gel electrophoresis, “DHPLC” denaturing high performance liquid chromatography, “qPCR” quantitative polymerase chain reaction. In reports including multiple families, those families with combined somatic and germ line mutation are identified as numbered in the original publications (e.g., Rubinstein-Taybi, Duchenne muscular dystrophy). * Mutant allele frequency has a theoretical maximum of 50% in heterozygous cells (100% in cells of hemizygous non-pseudo-autosomal X or Y linked males). Some reports refer to proportion of mutation-carrying cells, with a maximum of 100%; here these are corrected to the mutant allele frequency.

Germ line mosaicism can be similarly assessed by direct sequencing or genotyping, most easily through a sperm sample in males [83,84,85]. Germline samples may also be obtained through testicular aspiration in males or in ova collected from women after superovulation for *in vitro* fertilization. Some studies have also been performed on gonadal specimens obtained for diagnostic purposes by surgery or biopsy. Indirect assessment of the parental germ line can be performed by haplotype analysis of the children. For this approach, families with one or more children who carry the same mutation that does not appear to be present in either parent are required. One of the parents in such families is almost certainly mosaic for an apparent offspring *de novo* mutation, but further studies are needed to determine the presence and proportion of mutation-carrying cells in that parents’ germ line. Even if a parent’s germ line is fully mutant, only 50% of progeny, on average, would receive the mutant allele, as only half of gametes will carry the mutation; thus the occurrence of both affected and unaffected offspring alone is insufficient to demonstrate mosaicism of parental germ line cells. In order to establish germ line mosaicism in a parent, genotyping of markers surrounding the mutation must demonstrate that one particular grandparental haplotype present in the parental germ line carries a normal allele in one child and a mutant allele in another child. In the past, microsatellite markers were usually employed for such haplotyping, although SNP markers are equally applicable. The markers should be no more than a few centimorgans from the site of the mutation to minimize the possibility that recombination has occurred between the markers and the variant of interest. Alternatively, if an informative haplotype-defining inherited SNP is close enough to the *de novo* variant to be detectable in the same long-read sequencing amplicon, phase can be inferred directly.

Haplotyping is easier when the offspring are male and the variant of interest is on the non-pseudo-autosomal X-chromosome, as the offspring are hemizygous and haplotype-defining marker phase can be determined directly from their genotypes. Mosaicism for X-linked variants by haplotyping male offspring can only assess the maternal germ line of course. Haplotyping of autosomal variants can be performed to test mosaicism in both maternal and paternal germ lines.

## 5. Examples of Combined Somatic and Germ Line Mosaicism

A number of investigations have demonstrated that in mammals, including humans, somatic mosaicism frequently accompanies germ line mosaicism (Figure 1D and Figure 2D). Studies of XX↔XY chimaeric (“tetraparental”) mice, in which both XX and XY cells are present in most, if not all, somatic tissues, have shown the presence of both XX- and XY-germ cell precursors in the gonads of individual animals [86,87]. In humans, several cases have been reported in which low-level mosaicism for trisomy 21 in the blood and skin was found among the mothers of foetuses or infants with trisomy 21, implying concurrent germ line and somatic mosaicism [88].

Recurrent *de novo* disease-causing point mutations have been reported in many different autosomal dominant or X-linked conditions, in which germ line mosaicism was inferred because two or more children carried the same apparently *de novo* pathogenic mutation that could not be demonstrated by Sanger sequencing of leukocyte DNA from either parent. Typically other parental tissues such as hair follicles or skin fibroblasts were not examined, nor was leukocyte DNA deep-sequenced using next-generation methods. Thus as noted previously, the possibility of somatic mosaicism was not rigorously excluded. In fact a substantial proportion of such cases probably did involve parental somatic as well as germ line mosaicism.

Nevertheless, the genetics literature does contain many convincing examples of individuals with mosaicism for the same *de novo* mutation in both the germ line and somatic tissues (Table 1). We searched PubMed for references with the terms such as “somatic [and] germ line mosaicism”, and reviewed all reports that seemed directly relevant. In these reports, a variety of techniques were used to assess both somatic and germ line mosaicism, and a number of different genes were studied, including X, Y and autosomal genes. All of the cases were ascertained because a genetic disorder was found in association with an unexpected family history. These results are not contingent on any particular molecular experimental approach, gene or chromosome. The overrepresentation of some genes such as *COL1A1*, *COL1A2* and *DMD* is attributable to historical reasons or technical feasibility (X-linkage). Table 1 includes some examples where combined mosaicism is likely but not strictly proved for both somatic and germ line tissues. There is also one interesting example of Y-chromosome combined mosaicism for a variant in the male-determining *SRY* gene. It should be noted that the individual exhibiting the mosaic pattern of interest is not usually the ascertained proband, but is typically a parent (or grandparent). In some but not all cases the mosaic parent exhibits clinical symptoms, often mild and sometimes correlating with the degree of mutation mosaicism in the soma of that parent.

The significant number of these reports is consistent with the existence of multiple FGCs, and not with clonal origin of all germ cells from a single precursor that gives rise only to germ line progeny. The observation of combined somatic and germ line mosaicism, and its interpretation as evidence for non-clonality in the germ line, was previously noted by Zlotogora [89]. That review was not specifically focused on this issue, however, and only a few relevant literature examples were cited, while some other consistent cases were cited but not discussed.

Several authors in these reports comment that the aspect of mosaicism confused assignment of some families as to dominant *versus* recessive, or X-linked *versus* autosomal transmission. The occurrence of mutations in a subset of cells in unaffected carrier mosaics, either somatically or in their germ line, in some cases led to atypical transmission patterns. The authors warn clinical geneticists that mosaicism can also lead to incorrect estimates of recurrence risk of disease in families, or incorrect assessment of carrier status in individuals.

## 6. Number and Time of Specification of FGCs

In humans, the inner cell mass, which contains the developing embryo as well as some extraembryonic tissues, arises during days 4–5 post-fertilization; day 5 blastocysts contain from 10 to 50 cells resulting from up to six mitotic cycles [90]. It seems likely that FGC specification occurs later than this first stage of post-zygotic cell determination. PGCs are first observed in older embryos. If FGC specification occurs shortly before PGCs are detected, or if FGCs differentiate directly into PGCs, then more than 20 mitotic cycles could have taken place, making it much more likely that two different FGCs would have different ancestral cells at the time of a zygotic mutation event. These scenarios might be distinguished if the relative proportion of combined somatic plus germ line mosaicism (Figure 1D) could be determined relative to the other mosaicism patterns in a sufficiently large collection of families [17]. In one study of 288 cases of Duchenne muscular dystrophy, a *de novo* causal mutation was detected in 42 families [64]. In these 42, there were six families with multiple transmissions from an unaffected parent, implying mosaicism at least in the germ line. Somatic mosaicism was also detected in one germ line mosaic parent in these six families. In another study of 405 cases of retinoblastoma, a causal mutation was identified in the RB gene in 156 families [78]. Of these, 15 (10%) were shown to be mosaic, in the proband in nine cases (seven affected bilaterally, two affected unilaterally), and in an unaffected parent in six cases. It was noted that this represents an underestimate, as DNA from all family members was not available for the requisite analyses in all 156 families. Of the six cases where a mutation was inherited from a parent, in three cases the mutation was found in parental blood leukocytes at less than the expected 50%. As other tissues were not tested, nor was deep resequencing yet available, conceivably some of the other three cases may have also involved parental somatic mosaics. It remains to be seen whether similar statistics are obtained for other large ascertainments in which the requisite studies are performed, but these results suggest that combined somatic and germ line mutation is relatively common among families with some evidence of mosaicism. It should also be noted in passing that although many of the reported studies involve dominant conditions, this is partly due to a bias in clinical ascertainment. It is certainly the case that families with recessive genetic transmission can also involve mosaicism, where one allele is present in unaffected carriers, and the second, leading to the appearance of the recessive phenotype, arises *de novo*, either in a parent or in the proband. The second, *de novo* mutation, could also involve combined somatic plus germ line or only germ line parental mosaicism.

One alternative scenario which could lead to mosaicism of both soma and germ line in the case of a single clonal origin FGC, is if mitotic cells of the germ line were able to revert to a somatic fate, or if FGCs could yield both somatic and germ line descendants (in which case they would not really be FGCs by definition). Such developmental plasticity or de-differentiation is not the same as reversion of the causal mutation itself, which is very unlikely to occur in cells of the originally mutated individual. It is unclear whether cellular reversion to a somatic fate is feasible for the germ line, leaving multiple FGCs as the most likely explanation for the combined germ line and somatic mosaicism scenario. Other mutational events which could give rise to the observed pattern of combined somatic and germ line mosacism, in the presence of germ line clonality, include the same precise mutation arising independently in two different embryonic cells precursor to either the germ line or soma, or the precise reversion of a single mutation in some descendants of the original cell in which it arose. Recurrent mutation, and reversion events have been observed in different individuals in the entire human population, but the substantial number of examples of combined mosaicism reported here, in multiple genetic disorders, makes these multiple mutation scenarios occurring repeatedly in single individuals, exceedingly unlikely. Non-clonality of the germ line is a much more likely interpretation of the observations.

## 7. Use of High Throughput Sequencing for Developmental Lineage Studies in Humans

The theory behind this approach to analyze developmental cell specification is not specific to the germ line. Post-zygotic new mutations provide cell markers that can be used to trace lineages for any tissue [17]. In a recent example, mosaicism of a *GNAS1* mutation in a patient with McCune-Albright syndrome was documented in multiple tissues, both somatic and testicular [91]. Much recent work in cancer genomics employs next-generation sequencing to study tumour clonality, although that work is beyond the scope of this review. The difficulty of identifying embryonic mutations in the absence of a medical ascertainment means that large numbers of individuals (presumably post-mortem) would have to be sequenced to high coverage in multiple tissue types in order to identify and study the lineage patterns of such mutations. This approach has been demonstrated in mice, using whole genome sequencing of 25 different clonal cell lines originating from four different tissues of healthy animals [92]. 35 somatic, likely early embryonic mutations were identified and could be used to develop cell lineages in some cases pre-dating separation of the three germ layers during gastrulation. A somatic mutation rate of 1–2 substitutions per cell division was also determined, at least during embryogenesis. This rate seems higher than expected given the small number of observed genomic substitutions between monozygotic human twins; possibly the human studies suffer from a systematic bias against mutation detection given the technology and informatics approaches currently available. Developmental lineage studies are also being explored in humans, particularly in a medical context [93].

## 8. Conclusions

Genetic mosaics provide information about cellular lineages that is otherwise difficult to obtain, especially in humans. *De novo* mutations act as cell markers, allowing the tracing of developmental trajectories of all descendants of the cell in which the new mutation arises. Here we review the medical genetics literature and document many examples of simultaneous mosaicism for point mutations in both the some and germ line of individuals (typically the parents of medically ascertained probands). The most likely interpretation of these observations is that the human germ line is not clonal, *i.e.* adult mitotic germ line cells derive from multiple embryonic precursors, at least some of whom also have somatic cell descendants. This is consistent with mouse embryonic histological studies, and has important implications for human germ line development and also for medical geneticists attempting to estimate familial recurrence risks for genetic disorders. Rates of post-fertilization mutation are low, but with the advent of next-generation sequencing it should be possible to study similar lineage relationships among human tissues and organs without a preliminary medical ascertainment, by resequencing genomes of multiple tissue types from multiple individuals.

## References

[1] De Felici M., Coticchio C., Albertini D.F., de Santis L. (2013). Origin, Migration, and Proliferation of Human Primordial Germ Cells. Oogenesis.

[2] McKay D.G., Hertig A.T., Adams E.C., Danziger S. (1953). Histochemical observations on the germ cells of human embryos. Anat. Rec..

[3] Chiquoine A.D. (1954). The identification, origin, and migration of the primordial germ cells in the mouse embryo. Anat. Rec..

[4] Eddy E.M., Clark J.M., Gong D., Fenderson B.A. (1981). Origin and migration of primordial germ cells in mammals. Gamete Res..

[5] Hardisty M.W. (1967). The numbers of vertebrate primordial germ cells. Biol. Rev. Camb. Philos. Soc..

[6] O’Rahilly R., Muller F., Washington C.I.O. (1987). Developmental Stages in Human Embryos.

[7] Ginsburg M., Snow M.H., McLaren A. (1990). Primordial germ cells in the mouse embryo during gastrulation. Development.

[8] Fujimoto T., Miyayama Y., Fuyuta M. (1977). The origin, migration and fine morphology of human primordial germ cells. Anat. Rec..

[9] Pereda J., Zorn T., Soto-Suazo M. (2006). Migration of human and mouse primordial germ cells and colonization of the developing ovary: An ultrastructural and cytochemical study. Microsc. Res. Tech..

[10] De Felici M. (2004). Experimental approaches to the study of primordial germ cell lineage and proliferation. Hum. Reprod. Update.

[11] Anderson R.A., Fulton N., Cowan G., Coutts S., Saunders P.T. (2007). Conserved and divergent patterns of expression of DAZl, VASA and OCT4 in the germ cells of the human fetal ovary and testis. BMC dev. Biol..

[12] Diedrichs F., Mlody B., Matz P., Fuchs H., Chavez L., Drews K., Adjaye J. (2012). Comparative molecular portraits of human unfertilized oocytes and primordial germ cells at 10 weeks of gestation. Int. J. Dev. Biol..

[13] Castrillon D.H., Quade B.J., Wang T.Y., Quigley C., Crum C.P. (2000). The human VASA gene is specifically expressed in the germ cell lineage. Proc. Natl. Acad. Sci. USA.

[14] Perrett R.M., Turnpenny L., Eckert J.J., O’Shea M., Sonne S.B., Cameron I.T., Wilson D.I., Rajpert-de Meyts E., Hanley N.A. (2008). The early human germ cell lineage does not express SOX2 during *in vivo* development or upon *in vitro* culture. Biol. Reprod..

[15] De Felici M. (2009). Primordial germ cell biology at the beginning of the xxi century. Int. J. Dev. Biol..

[16] Zheng C.J., Luebeck E.G., Byers B., Moolgavkar S.H. (2005). On the number of founding germ cells in humans. Theor. Biol. Med. Model..

[17] Nesbitt M.N., Gartler S.M. (1971). The applications of genetic mosaicism to developmental problems. Annu. Rev. genet..

[18] Biesecker L.G., Spinner N.B. (2013). A genomic view of mosaicism and human disease. Nat. Rev. Genet..

[19] Erickson R.P. (2014). Recent advances in the study of somatic mosaicism and diseases other than cancer. Curr. Opin. Genet. Dev..

[20] Lupski J.R. (2013). Genome mosaicism—One human, multiple genomes. Science.

[21] Erickson R.P. (2010). Somatic gene mutation and human disease other than cancer: An update. Mutat. Res..

[22] Drost J.B., Lee W.R. (1998). The developmental basis for germline mosaicism in mouse and drosophila melanogaster. Genetica.

[23] Yu N., Kruskall M.S., Yunis J.J., Knoll J.H., Uhl L., Alosco S., Ohashi M., Clavijo O., Husain Z., Yunis E.J. (2002). Disputed maternity leading to identification of tetragametic chimerism. N. Engl. J. Med..

[24] Yu Q., Li Q., Gao S., Su Y., Deng Z. (2011). Congenital tetragametic blood chimerism explains a case of questionable paternity. J. Forensic Sci..

[25] Dal G.M., Erguner B., Sagiroglu M.S., Yuksel B., Onat O.E., Alkan C., Ozcelik T. (2014). Early postzygotic mutations contribute to *de novo* variation in a healthy monozygotic twin pair. J. Med. Genet..

[26] Roach J.C., Glusman G., Smit A.F., Huff C.D., Hubley R., Shannon P.T., Rowen L., Pant K.P., Goodman N., Bamshad M. (2010). Analysis of genetic inheritance in a family quartet by whole-genome sequencing. Science.

[27] Awadalla P., Gauthier J., Myers R.A., Casals F., Hamdan F.F., Griffing A.R., Cote M., Henrion E., Spiegelman D., Tarabeux J. (2010). Direct measure of the *de novo* mutation rate in autism and schizophrenia cohorts. Am. J. Hum. Genet..

[28] Conrad D.F., Keebler J.E., DePristo M.A., Lindsay S.J., Zhang Y., Casals F., Idaghdour Y., Hartl C.L., Torroja C., Garimella K.V. (2011). Variation in genome-wide mutation rates within and between human families. Nat. Genet..

[29] Michaelson J.J., Shi Y., Gujral M., Zheng H., Malhotra D., Jin X., Jian M., Liu G., Greer D., Bhandari A. (2012). Whole-genome sequencing in autism identifies hot spots for *de novo* germline mutation. Cell.

[30] Wang H., Zhu X. (2014). *De novo* mutations discovered in 8 mexican american families through whole genome sequencing. BMC Proc..

[31] Campbell C.D., Eichler E.E. (2013). Properties and rates of germline mutations in humans. Trends genet. TIG.

[32] Silva S., Martins Y., Matias A., Blickstein I. (2011). Why are monozygotic twins different?. J. Perinat. Med..

[33] Baranzini S.E., Mudge J., van Velkinburgh J.C., Khankhanian P., Khrebtukova I., Miller N.A., Zhang L., Farmer A.D., Bell C.J., Kim R.W. (2010). Genome, epigenome and rna sequences of monozygotic twins discordant for multiple sclerosis. Nature.

[34] Chaiyasap P., Kulawonganunchai S., Srichomthong C., Tongsima S., Suphapeetiporn K., Shotelersuk V. (2014). Whole genome and exome sequencing of monozygotic twins with trisomy 21, discordant for a congenital heart defect and epilepsy. PLOS ONE.

[35] Furukawa H., Oka S., Matsui T., Hashimoto A., Arinuma Y., Komiya A., Fukui N., Tsuchiya N., Tohma S. (2013). Genome, epigenome and transcriptome analyses of a pair of monozygotic twins discordant for systemic lupus erythematosus. Hum. Immunol..

[36] Jin M., Zhu S., Hu P., Liu D., Li Q., Li Z., Zhang X., Xie Y., Chen X. (2014). Genomic and epigenomic analyses of monozygotic twins discordant for congenital renal agenesis. Am. J. Kidney Dis..

[37] Kondo S., Schutte B.C., Richardson R.J., Bjork B.C., Knight A.S., Watanabe Y., Howard E., de Lima R.L., Daack-Hirsch S., Sander A. (2002). Mutations in IRF6 cause van der woude and popliteal pterygium syndromes. Nat. Genet..

[38] Magne F., Serpa R., van Vliet G., Samuels M.E., Deladoey J. (2015). Somatic mutations are not observed by exome sequencing of lymphocyte DNA from monozygotic twins discordant for congenital hypothyroidism due to thyroid dysgenesis. Horm. Res. Paediatr..

[39] Petersen B.S., Spehlmann M.E., Raedler A., Stade B., Thomsen I., Rabionet R., Rosenstiel P., Schreiber S., Franke A. (2014). Whole genome and exome sequencing of monozygotic twins discordant for Crohn’s disease. BMC Genom..

[40] Reumers J., de Rijk P., Zhao H., Liekens A., Smeets D., Cleary J., van Loo P., van Den Bossche M., Catthoor K., Sabbe B. (2012). Optimized filtering reduces the error rate in detecting genomic variants by short-read sequencing. Nat. Biotechnol..

[41] Weber-Lehmann J., Schilling E., Gradl G., Richter D.C., Wiehler J., Rolf B. (2014). Finding the needle in the haystack: Differentiating “identical” twins in paternity testing and forensics by ultra-deep next generation sequencing. Forensic Sci. Int. Genet..

[42] Li R., Montpetit A., Rousseau M., Wu S.Y., Greenwood C.M., Spector T.D., Pollak M., Polychronakos C., Richards J.B. (2014). Somatic point mutations occurring early in development: A monozygotic twin study. J. Med. Genet..

[43] Kondrashov A.S. (2012). Genetics: The rate of human mutation. Nature.

[44] Kondrashov A.S. (2003). Direct estimates of human per nucleotide mutation rates at 20 loci causing mendelian diseases. Hum. Mutat..

[45] Samuels M.E. (2010). Saturation of the human phenome. Curr. Genom..

[46] Huang A.Y., Xu X., Ye A.Y., Wu Q., Yan L., Zhao B., Yang X., He Y., Wang S., Zhang Z. (2014). Postzygotic single-nucleotide mosaicisms in whole-genome sequences of clinically unremarkable individuals. Cell Res..

[47] Lindhurst M.J., Sapp J.C., Teer J.K., Johnston J.J., Finn E.M., Peters K., Turner J., Cannons J.L., Bick D., Blakemore L. (2011). A mosaic activating mutation in AKT1 associated with the proteus syndrome. N. Engl. J. med..

[48] Marsh D.J., Trahair T.N., Kirk E.P. (2011). Mutant AKT1 in proteus syndrome. N. Engl. J. Med..

[49] Schwindinger W.F., Francomano C.A., Levine M.A. (1992). Identification of a mutation in the gene encoding the alpha subunit of the stimulatory g protein of adenylyl cyclase in mccune-albright syndrome. Proc. Natl. Acad. Sci. USA.

[50] Weinstein L.S., Shenker A., Gejman P.V., Merino M.J., Friedman E., Spiegel A.M. (1991). Activating mutations of the stimulatory g protein in the mccune-albright syndrome. N. Engl. J. Med..

[51] Choi S.K., Yoon S.R., Calabrese P., Arnheim N. (2008). A germ-line-selective advantage rather than an increased mutation rate can explain some unexpectedly common human disease mutations. Proc. Natl. Acad. Sci. USA.

[52] Choi S.K., Yoon S.R., Calabrese P., Arnheim N. (2012). Positive selection for new disease mutations in the human germline: Evidence from the heritable cancer syndrome multiple endocrine neoplasia type 2B. PLOS Genet..

[53] Goriely A., McVean G.A., Rojmyr M., Ingemarsson B., Wilkie A.O. (2003). Evidence for selective advantage of pathogenic FGFR2 mutations in the male germ line. Science.

[54] Yoon S.R., Choi S.K., Eboreime J., Gelb B.D., Calabrese P., Arnheim N. (2013). Age-dependent germline mosaicism of the most common noonan syndrome mutation shows the signature of germline selection. Am. J. Hum. Genet..

[55] Ducamp S., Schneider-Yin X., de Rooij F., Clayton J., Fratz E.J., Rudd A., Ostapowicz G., Varigos G., Lefebvre T., Deybach J.C. (2013). Molecular and functional analysis of the C-terminal region of human erythroid-specific 5-aminolevulinic synthase associated with X-linked dominant protoporphyria (XLDPP). Hum. Mol. Genet..

[56] Boehmer A.L., Brinkmann A.O., Niermeijer M.F., Bakker L., Halley D.J., Drop S.L. (1997). Germ-line and somatic mosaicism in the androgen insensitivity syndrome: Implications for genetic counseling. Am. J. Hum. Genet..

[57] Cohn D.H., Starman B.J., Blumberg B., Byers P.H. (1990). Recurrence of lethal osteogenesis imperfecta due to parental mosaicism for a dominant mutation in a human type I collagen gene (COL1A1). Am. J. Hum. Genet..

[58] Wallis G.A., Starman B.J., Zinn A.B., Byers P.H. (1990). Variable expression of osteogenesis imperfecta in a nuclear family is explained by somatic mosaicism for a lethal point mutation in the alpha 1(I) gene (COL1A1) of type I collagen in a parent. Am. J. Hum. Genet..

[59] Namikawa C., Suzumori K., Fukushima Y., Sasaki M., Hata A. (1995). Recurrence of osteogenesis imperfecta because of paternal mosaicism: Gly862-->ser substitution in a type I collagen gene (COL1A1). Hum. Genet..

[60] Edwards M.J., Wenstrup R.J., Byers P.H., Cohn D.H. (1992). Recurrence of lethal osteogenesis imperfecta due to parental mosaicism for a mutation in the COL1A2 gene of type I collagen. The mosaic parent exhibits phenotypic features of a mild form of the disease. Hum. Mutat..

[61] Lund A.M., Schwartz M., Raghunath M., Steinmann B., Skovby F. (1996). Gly802asp substitution in the pro alpha 2(I) collagen chain in a family with recurrent osteogenesis imperfecta due to paternal mosaicism. Eur. J. Hum. Genet..

[62] Chiang P.W., Lee N.C., Chien N., Hwu W.L., Spector E., Tsai A.C. (2009). Somatic and germ-line mosaicism in rubinstein-taybi syndrome. Am. J. Med. Genet. A.

[63] Vulliamy T.J., Knight S.W., Heiss N.S., Smith O.P., Poustka A., Dokal I., Mason P.J. (1999). Dyskeratosis congenita caused by a 3' deletion: Germline and somatic mosaicism in a female carrier. Blood.

[64] Bakker E., Veenema H., den Dunnen J.T., van Broeckhoven C., Grootscholten P.M., Bonten E.J., van Ommen G.J., Pearson P.L. (1989). Germinal mosaicism increases the recurrence risk for ‘new’ duchenne muscular dystrophy mutations. J. Med. Genet..

[65] Bunyan D.J., Robinson D.O., Collins A.L., Cockwell A.E., Bullman H.M., Whittaker P.A. (1994). Germline and somatic mosaicism in a female carrier of duchenne muscular dystrophy. Hum. Genet..

[66] Cutler J.A., Mitchell M.J., Smith M.P., Savidge G.F. (2004). Germline mosaicism resulting in the transmission of severe hemophilia b from a grandfather with a mild deficiency. Am. J. Med. genet. A.

[67] Kohler J., Rupilius B., Otto M., Bathke K., Koch M.C. (1996). Germline mosaicism in 4q35 facioscapulohumeral muscular dystrophy (FSHD1A) occurring predominantly in oogenesis. Hum. Genet..

[68] Gitschier J. (1988). Maternal duplication associated with gene deletion in sporadic hemophilia. Am. J. Hum. Genet..

[69] Casey G.J., Rodgers S.E., Hall J.R., Rudzki Z., Lloyd J.V. (1999). Grandpaternal mosaicism in a family with isolated haemophilia A. Br. J. Haematol..

[70] Willers I. (2004). Germline mosaicism complicates molecular diagnosis of lesch-nyhan syndrome. Prenat. Diagn..

[71] Froissart R., Maire I., Bonnet V., Levade T., Bozon D. (1997). Germline and somatic mosaicism in a female carrier of hunter disease. J. Med. Genet..

[72] Vits L., Chitayat D., van Camp G., Holden J.J., Fransen E., Willems P.J. (1998). Evidence for somatic and germline mosaicism in crash syndrome. Hum. Mutat..

[73] Bijlsma E.K., Wallace A.J., Evans D.G. (1997). Misleading linkage results in an NF2 presymptomatic test owing to mosaicism. J. Med. Genet..

[74] Satre V., Monnier N., Berthoin F., Ayuso C., Joannard A., Jouk P.S., Lopez-Pajares I., Megabarne A., Philippe H.J., Plauchu H. (1999). Characterization of a germline mosaicism in families with lowe syndrome, and identification of seven novel mutations in the OCRL1 gene. Am. J. Hum. Genet..

[75] Goji K., Ozaki K., Sadewa A.H., Nishio H., Matsuo M. (2006). Somatic and germline mosaicism for a mutation of the phex gene can lead to genetic transmission of x-linked hypophosphatemic rickets that mimics an autosomal dominant trait. J. Clin. Endocrinol. Metab..

[76] Tan A.Y., Blumenfeld J., Michaeel A., Donahue S., Bobb W., Parker T., Levine D., Rennert H. (2015). Autosomal dominant polycystic kidney disease caused by somatic and germline mosaicism. Clin. Genet..

[77] Beck J.A., Poulter M., Campbell T.A., Uphill J.B., Adamson G., Geddes J.F., Revesz T., Davis M.B., Wood N.W., Collinge J. (2004). Somatic and germline mosaicism in sporadic early-onset alzheimer’s disease. Hum. Mol. Genet..

[78] Sippel K.C., Fraioli R.E., Smith G.D., Schalkoff M.E., Sutherland J., Gallie B.L., Dryja T.P. (1998). Frequency of somatic and germ-line mosaicism in retinoblastoma: Implications for genetic counseling. Am. J. Hum. Genet..

[79] Munier F.L., Thonney F., Girardet A., Balmer A., Claustre M., Pellestor F., Senn A., Pescia G., Schorderet D.F. (1998). Evidence of somatic and germinal mosaicism in pseudo-low-penetrant hereditary retinoblastoma, by constitutional and single-sperm mutation analysis. Am. J. Hum. Genet..

[80] Eggermann T., Zerres K., Anhuf D., Kotzot D., Fauth C., Rudnik-Schoneborn S. (2005). Somatic mosaicism for a heterozygous deletion of the survival motor neuron (SMN1) gene. Eur. J. Hum. Genet..

[81] Faivre L., Williamson K.A., Faber V., Laurent N., Grimaldi M., Thauvin-Robinet C., Durand C., Mugneret F., Gouyon J.B., Bron A. (2006). Recurrence of SOX2 anophthalmia syndrome with gonosomal mosaicism in a phenotypically normal mother. Am. J. Med. Genet. A.

[82] Hines R.S., Tho S.P., Zhang Y.Y., Plouffe L., Hansen K.A., Khan I., McDonough P.G. (1997). Paternal somatic and germ-line mosaicism for a sex-determining region on Y (SRY) missense mutation leading to recurrent 46,XY sex reversal. Fertil. Steril..

[83] Dakouane Giudicelli M., Serazin V., le Sciellour C.R., Albert M., Selva J., Giudicelli Y. (2008). Increased achondroplasia mutation frequency with advanced age and evidence for G1138A mosaicism in human testis biopsies. Fertil. Steril..

[84] Rey R.A., Venara M., Coutant R., Trabut J.B., Rouleau S., Lahlou N., Sultan C., Limal J.M., Picard J.Y., Lumbroso S. (2006). Unexpected mosaicism of R201H-GNAS1 mutant-bearing cells in the testes underlie macro-orchidism without sexual precocity in mccune-albright syndrome. Hum. Mol. Genet..

[85] Qin J., Calabrese P., Tiemann-Boege I., Shinde D.N., Yoon S.R., Gelfand D., Bauer K., Arnheim N. (2007). The molecular anatomy of spontaneous germline mutations in human testes. PLOS Biol..

[86] Palmer S.J., Burgoyne P.S. (1991). *In situ* analysis of fetal, prepuberal and adult xx----xy chimaeric mouse testes: Sertoli cells are predominantly, but not exclusively, XY. Development.

[87] Warr N., Greenfield A. (2012). The molecular and cellular basis of gonadal sex reversal in mice and humans. Wiley Interdiscipl. Rev. Dev. Biol..

[88] Hulten M.A., Patel S.D., Tankimanova M., Westgren M., Papadogiannakis N., Jonsson A.M., Iwarsson E. (2008). On the origin of trisomy 21 down syndrome. Mol. Cytogenet..

[89] Zlotogora J. (1998). Germ line mosaicism. Hum. Genet..

[90] Herbert M., Wolstenholme J., Murdoch A.P., Butler T.J. (1995). Mitotic activity during preimplantation development of human embryos. J. Reprod. Fertil..

[91] Vasilev V., Daly A.F., Thiry A., Petrossians P., Fina F., Rostomyan L., Silvy M., Enjalbert A., Barlier A., Beckers A. (2014). Mccune-albright syndrome: A detailed pathological and genetic analysis of disease effects in an adult patient. J. Clin. Endocrinol. Metab..

[92] Behjati S., Huch M., van Boxtel R., Karthaus W., Wedge D.C., Tamuri A.U., Martincorena I., Petljak M., Alexandrov L.B., Gundem G. (2014). Genome sequencing of normal cells reveals developmental lineages and mutational processes. Nature.

[93] Oetting W.S., Greenblatt M.S., Brookes A.J., Karchin R., Mooney S.D. (2015). Germline & somatic mosaicism: The 2014 annual scientific meeting of the human genome variation society. Hum. Mutat..

